# Was the Risk from Nursing-Home Evacuation after the Fukushima Accident Higher than the Radiation Risk?

**DOI:** 10.1371/journal.pone.0137906

**Published:** 2015-09-11

**Authors:** Michio Murakami, Kyoko Ono, Masaharu Tsubokura, Shuhei Nomura, Tomoyoshi Oikawa, Tosihiro Oka, Masahiro Kami, Taikan Oki

**Affiliations:** 1 Institute of Industrial Science, The University of Tokyo, 4-6-1 Komaba, Meguro, Tokyo, 153–8505, Japan; 2 Research Institute of Science for Safety and Sustainability, National Institute of Advanced Industrial Science and Technology (AIST), 16–1, Onogawa, Tsukuba, 305–8569, Japan; 3 Division of Social Communication System for Advanced Clinical Research, The Institute of Medical Science, The University of Tokyo, 4-6-1, Shirokanedai, Minato, Tokyo, 108–8639, Japan; 4 Department of Epidemiology and Biostatistics, School of Public Health, Imperial College London, Norfolk Place, London W2 1PG, United Kingdom; 5 Department of Radiation Protection, Minamisoma Municipal General Hospital, 2-54-6 Takami, Haramachi, Minamisoma, Fukushima, 975–0033, Japan; 6 Faculty of Economics, Fukui Prefectural University, 4-1-1, Matsuoka-Kenjojima, Eiheiji-Town, Yoshida County, Fukui, 910–1195, Japan; University of Zurich, SWITZERLAND

## Abstract

After the 2011 accident at the Fukushima Daiichi nuclear power plant, nursing-home residents and staff were evacuated voluntarily from damaged areas to avoid radiation exposure. Unfortunately, the evacuation resulted in increased mortalities among nursing home residents. We assessed the risk trade-off between evacuation and radiation for 191 residents and 184 staff at three nursing homes by using the same detriment indicator, namely loss of life expectancy (LLE), under four scenarios, i.e. “rapid evacuation (in accordance with the actual situation; i.e. evacuation on 22 March),” “deliberate evacuation (i.e. evacuation on 20 June),” “20-mSv exposure,” and “100-mSv exposure.” The LLE from evacuation-related mortality among nursing home residents was assessed with survival probability data from nursing homes in the city of Minamisoma and the city of Soma. The LLE from radiation mortality was calculated from the estimated age-specific mortality rates from leukemia and all solid cancers based on the additional effective doses and the survival probabilities. The total LLE of residents due to evacuation-related risks in rapid evacuation was 11,000 persons-d—much higher than the total LLEs of residents and staff due to radiation in the other scenarios (27, 1100, and 5800 persons-d for deliberate evacuation, 20 mSv-exposure, and 100 mSv-exposure, respectively). The latitude for reducing evacuation risks among nursing home residents is surprisingly large. Evacuation regulation and planning should therefore be well balanced with the trade-offs against radiation risks. This is the first quantitative assessment of the risk trade-off between radiation exposure and evacuation after a nuclear power plant accident.

## Introduction

The 2011 accident that occurred at the Tokyo Electric Power Company’s Fukushima Daiichi nuclear power plant after the Great East Japan Earthquake on 11 March created multiple social risks. Among these multiple risks, radiation-related health risks were of particular concern and have therefore been investigated well. To avoid radiation exposure of the public, on 12 March 2011 the Japanese government ordered a full evacuation of residents living within 20 km of the Fukushima Daiichi nuclear power plant and 10 km of the Fukushima Daini nuclear power plant. On 22 April it also ordered the evacuation within 1 month of residents with an additional effective dose of ≥20 mSv y^–1^. This value comes from the lowest reference level in the effective dose band for emergency exposure situations (20 to 100 mSv y^-1^), as determined by the International Commission on Radiological Protection (ICRP) [1]. Radiation doses from both external and internal exposure (inhalation and ingestion), and the consequent cancer risks, have been comprehensively evaluated [2–4].

However, evacuation-related risks are also a major issue. The number of deaths indirectly related to the earthquake in Fukushima Prefecture eventually rose to >1700 [5]. These deaths were considered to be associated mainly with the physical or mental stresses related to evacuee living [5]. Various metabolic profiles, including body mass index, waist circumference, hemoglobin A1c, and high-density lipoprotein cholesterol level, in residents living in temporary housing provided by local governments were impaired after the evacuation, and adverse health effects were a matter of concern [6]. Such evacuation-related risks have been conceptually and qualitatively known since the 1986 Chernobyl accident [7]; however, the risk trade-off between radiation and evacuation has not been evaluated owing to a lack of quantitative assessment of evacuation-related risks. The lowest reference level in the effective dose band for emergency exposure situations (20 mSv y^–1^), which was used as the evacuation criterion in Japan, originated from constraints set by the ICRP for occupational exposure [8], mainly by using the Royal Society’s acceptable risk values as a reference [9]. The ICRP uses the concept of optimization of protection, and the importance of quantitative assessment of risk trade-off is now increasing.

Evacuation has a critical impact on the weak. In the 2011 accident, the evacuation-related risk was especially serious in nursing-home residents in the city of Minamisoma, in Fukushima Prefecture. These nursing home facilities were located 20 to 30 km from the Fukushima Daiichi nuclear power plant. The Japanese government issued a directive to “shelter indoors” and designated these areas as a voluntary evacuation zone just after the accident; then, on 22 April 2011, the areas were re-designated as evacuation-prepared in case of emergency (S1 Fig in the Supporting Information (SI)). Although the areas were outside the compulsory evacuation zone, all nursing home residents and staff chose to be evacuated voluntarily within 2 weeks after the accident because of anxiety about radiation exposure and instability of the nuclear power plants, as well as a lack of resources such as medical drugs. Unfortunately, the evacuation resulted in increased mortalities among nursing home residents, as was recently revealed by our retrospective cohort survival survey [10]. This high mortality rate is attributable to multiple factors, and especially to the burden of the evacuation itself, changes in medical staff, and a lack of preparedness at the evacuation sites [11,12]. Unlike any increased radiation risk, the increased mortality rate due to evacuation might have been alleviated if the evacuation had been done slowly and deliberately after the nursing homes at the evacuation sites had prepared to care for their residents accordingly.

Our objective here was to assess evacuation-related risks and compare with radiation risks for nursing-home residents and staff. We also discussed the risk trade-off in emergency of a nuclear accident. We estimated the mortality risks to nursing-home residents and staff in the city of Minamisoma due to the evacuation and due to radiation risk by using the same detriment indicator, namely loss of life expectancy (LLE). We considered four scenarios, namely “rapid evacuation” (in accordance with the actual situation; i.e. evacuation on 22 March); “deliberate evacuation” (instead of immediate evacuation, slow evacuation after completion of preparations for hospitalization, i.e. evacuation on 20 June); “20-mSv exposure;” and “100-mSv exposure” (see “Scenarios” in Methods). Radiation risks were estimated from doses to the colon and bone marrow under the assumption that cancer mortality risks can be evaluated by using these organ doses (see details in “Estimation of cancer risks and LLE due to radiation” in Methods). To our knowledge, this is the first quantitative assessment of the risk trade-off between radiation exposure and evacuation after a nuclear power plant accident. The information obtained will be useful for developing guidelines and plans for evacuation in nuclear power plant accidents.

## Methods

### Study design and participants

Ethical approval for the study was granted by the ethics committee of the Institute of Medical Science, The University of Tokyo (authorization number 26-70-1128). We used data from three of the eight nursing home facilities in Minamisoma (Facilities 1, 2, and 4, as named in ref [10]) as Nursing home group A. These three nursing homes were selected on the basis of 1) the type of evacuation classification of the area in which they were located and 2) data availability, including data on the numbers of staff. All three facilities were located in the “shelter indoors” and voluntary evacuation zone and were specialized nursing homes for the elderly. Two other two facilities included in our previous study [10] were excluded; one facility was located outside the “shelter indoors” and voluntary evacuation zone and the other was of a different type, namely a healthcare facility for the elderly. The three facilities used here accounted for 76.9% of all residents in nursing homes specializing in the elderly and located in the “shelter indoors” and voluntary evacuation zone in Minamisoma. Data from two of the three nursing homes in the city of Soma (i.e. Nursing home group B) were used as controls; these facilities represented 62.5% of all nursing home residents in Soma. These two nursing homes were also selected on the basis of data availability. The locations of Minamisoma and Soma and the classification of areas for evacuation are shown in S1 Fig. The total numbers of nursing home residents before and after the disaster (11 March 2011) were 357 and 191, respectively (Nursing home group A) and 500 and 198 (Nursing home group B) (Table 1). “Nursing home residents before the disaster” represented those who had entered the nursing home before 11 March 2011 and comprised those who had died before the disaster, left the nursing home, or lived until the end of the disaster. “Nursing home residents after the disaster” represented those who had been in the nursing home on 11 March 2011 and comprised those who had died after the disaster, left the nursing home, or lived until the end of the survey. The total number of staff in Nursing home group A was 184 (Table 1).

**Table 1:** Characteristics of residents and staff in nursing homes. Values in parentheses represent numbers of nursing home residents on 11 March 2011. Nursing home group A was evacuated, whereas Nursing home group B was not.

	Nursing home group A	Nursing home group B
Nursing home residents		
Number (age at entry)		
Male		
40–49	0(0)	1(0)
50–59	1(1)	3(2)
60–69	17(12)	6(1)
70–79	20(8)	31(17)
80–89	38(18)	65(15)
90+	12(4)	15(2)
Total	88(43)	121(37)
Female		
40–49	0(0)	0(0)
50–59	3(3)	2(1)
60–69	13(4)	12(7)
70–79	60(40)	67(28)
80–89	133(75)	197(98)
90+	60(26)	101(27)
Total	269(148)	379(161)
Number (care level^a^)		
Low/moderate	181(126)	365(142)
High	176(65)	134(56)
Number of death	196^b^(60)	261^b^(85)
Person-years		
Pre-disaster	910	882
Post-disaster	118	332
Nursing home staff		
Number (age at disaster)		
Male		
19–29	15	
30–39	17	
40–49	7	
50–59	0	
60–69	3	
Total	42	
Female		
19–29	32	
30–39	42	
40–49	33	
50–59	27	
60–69	8	
Total	142	

^a^ Total of low/moderate and high care patients in Nursing home group B (499) did not equal the total number of nursing home residents (500) owing to lack of data.

^b^ Values represent the numbers of deaths before and after the disaster combined. The number of deaths before the disaster was 136 for Nursing home group A and 176 for Nursing home group B.

### Scenarios

To evaluate the risk trade-off between evacuation and radiation, we considered four scenarios: Scenario 1 was “rapid evacuation”. In accordance with the actual situation (i.e. evacuation that occurred on 22 March), nursing home residents and staff were assumed to have stayed in Minamisoma until 21 March and to have arrived at the evacuation site on 22 March. Under this scenario, the evacuation site was considered to be Kanagawa Prefecture, ~250 km from the Daiichi Nuclear Power Plant (S1 Fig), because 63.9% of nursing home residents were moved to this prefecture in the actual situation. Scenario 2 was deliberate evacuation. Under this scenario, we considered a “90-day delayed evacuation” (i.e. evacuation on 20 June). Nursing home residents and staff were assumed to have stayed in Minamisoma until 19 June and to have arrived at the evacuation site on 20 June. This assumed that the nursing homes at the sites to which the patients were evacuated were able to resume medical treatment with medical staff and resources on 20 June, because many other hospitals (e.g. Minamisoma Municipal General Hospital) restarted their hospitalizations on 20 June. The other two scenarios were non-evacuation with radiation exposures of 20 mSv or 100 mSv in the first year, which are the lowest and highest reference levels, respectively, in the ICRP effective dose bands for emergency exposure situations [1]. In these scenarios, nursing home residents and staff were assumed not to have been evacuated but to have had radiation exposures of 20 mSv or 100 mSv.

We assessed the risks of evacuation and radiation under these four scenarios. In general, to demonstrate the degree of evacuation risk, evacuation risks were calculated in such a way that they were not overestimated, whereas radiation risks were calculated such that they were not underestimated.

### Estimation of LLE due to evacuation

#### Outline

To assess the LLE from evacuation-related mortality among nursing home residents, we used survival probability data from three representative nursing homes in Nursing home group A and two in Nursing home group B (Table 1). Nursing home group B, from which no evacuations were conducted, was used as a control.

We calculated LLEs attributable to both evacuation- plus non-evacuation-related effects (e.g. disaster shock) and to non-evacuation-related effects alone. The former LLE was based on survival data for residents in Nursing home group A, and the latter was based on the data for residents in Nursing home group B.

**Fig 1:**
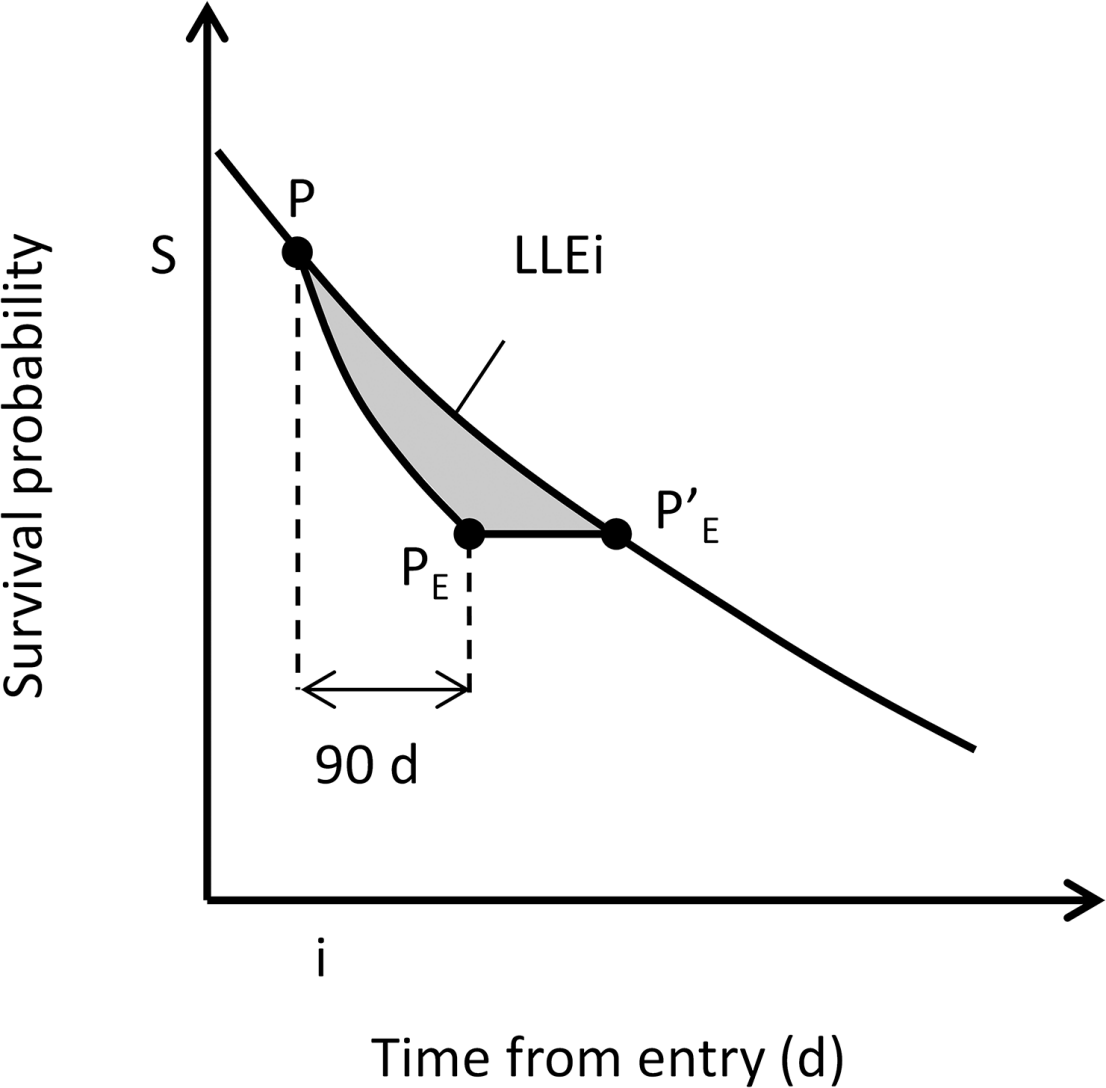
Conceptual diagrams for estimation of LLE of nursing home residents owing to evacuation or disaster shock, or both. Two slopes for survival reduction post- and pre-disaster were used to calculate the LLEs of nursing home residents so as not to overestimate the risk of evacuation or disaster shock, or both.

**Fig 2:**
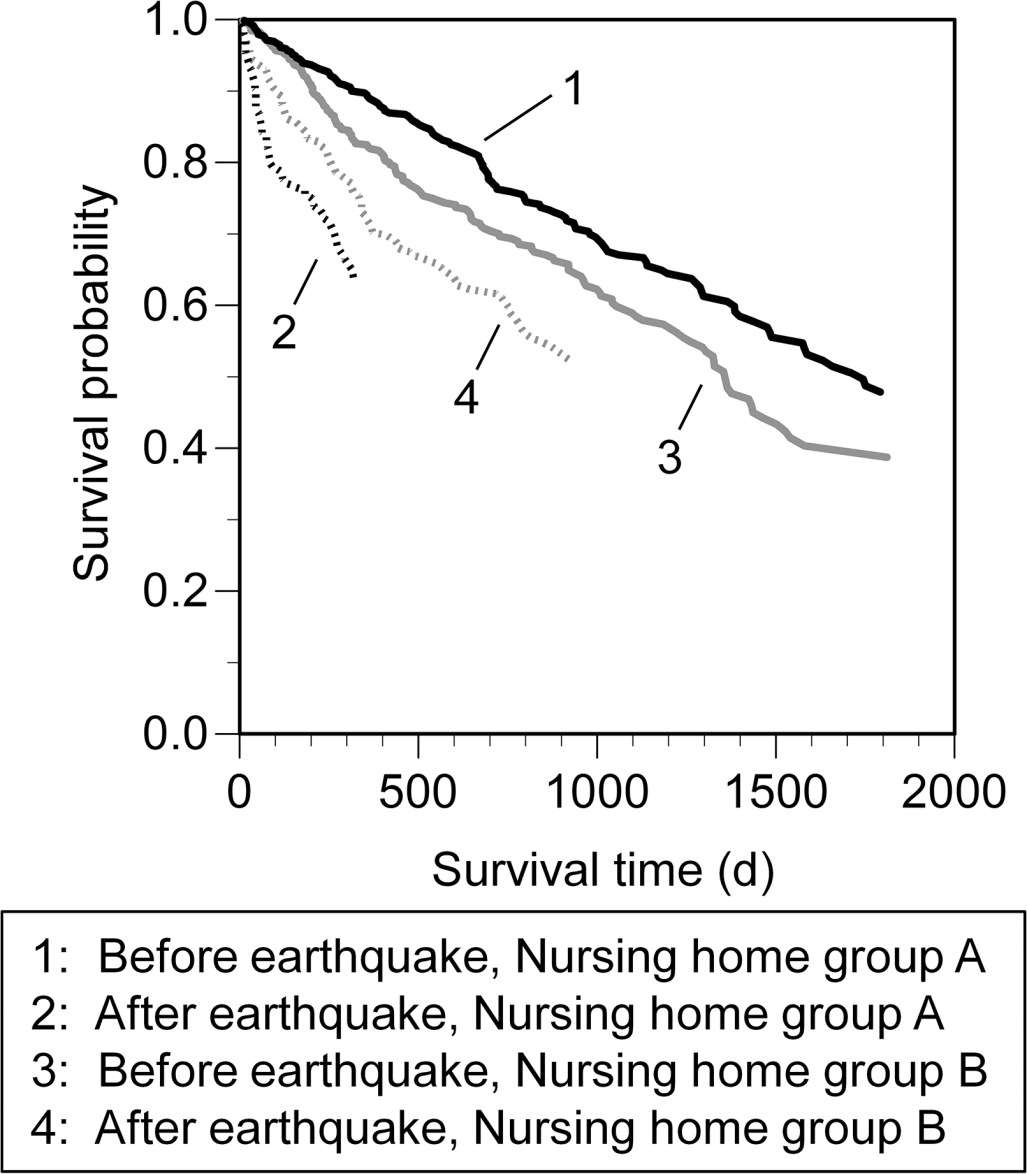
Estimated pre- and post-disaster survival in Nursing home groups A and B. The survival reductions post-disaster (within approximately 90 days after the disaster) were much higher than those pre-disaster in both cities, particularly in Nursing home group A, indicating that there was an increase in mortality risk due to evacuation. 1: y = 0.994–0.000293x (*r* ^2^ = 0.997, *P* < 0.001); 2: y = 0.999–0.00238x (x ≤ 90, *r* ^2^ = 0.986, *P* < 0.001); 3: y = 0.971–0.000357x (*r* ^2^ = 0.984, *P* < 0.001); 4: y = 0.985–0.000881x (x ≤ 90, *r* ^2^ = 0.920, *P* < 0.001).

LLE calculation required two survival curves—one an affected cohort (post-disaster) and the other a cohort without adverse effects (pre-disaster). LLE was obtained as the difference in the areas under the survival curve for an affected cohort and for an unaffected cohort (gray area, Fig 1). We regarded the pre-disaster survival curve as representing that for the unaffected cohort and the post-disaster survival curve (in the 90 days after the disaster) as representing that for the affected cohort. This is because sharp reductions in survival rate were observed in both Nursing home group A and Nursing home group B in the 90 days after the disaster (see “Evacuation-related mortality risks” in Results and Fig 2). The reduction in mortality rate eased after 90 days, although it did not recover to the pre-quake level. Thus LLEs calculated in this way can be regarded as being underestimated.

#### Calculation of survival probabilities

Survival probabilities were obtained for four cohorts, namely post- and pre-disaster in Nursing home groups A and B. These survival probabilities were adjusted by using the Cox proportional hazard regression model, and three confounding factors (i.e., age at entry, sex, and care level) were selected in accordance with a previous study [10]. We thus obtained a formula for the survival curve S(t) by cohort and by group.

$$S(t)=S_{0i}{\left(t\right)}^{\exp(\beta_{1}\cdot (X_{1}-{\bar{X}}_{1})+\beta_{2}\cdot (X_{2}-{\bar{X}}_{2})+\beta_{3}\cdot (X_{3}-{\bar{X}}_{3}))}$$ (Eq 1)

t (survival time) = 0 represents the time at entry in the case of the pre-disaster cohort; in the case of the post-disaster cohort it represents the time of the disaster. The confounding factors were categorized as follows: age at entry (X_1_), namely age 40–69 = 0, age 70–79 = 1, age 80–89 = 2, age 90+ = 3; care level (X_2_), namely low/moderate = 0, high = 1; and sex (X_3_), namely male = 0, female = 1. The arithmetic means of the confounding factors were as follows: ${\bar{X}}_{1}$, 2.16; ${\bar{X}}_{2}$, 0.35; and ${\bar{X}}_{3}$, 0.77. After the coefficients of the Cox proportional hazard regression model (β_1_, β_2_, and β_3_) had been obtained (S1 Table), S_0i_(t) was obtained as the fitted baseline survival probability for each cohort i (i.e., the groups categorized by location (Nursing home group A or B) and pre- or post-disaster) and was approximated as linear (see “Evacuation-related mortality risks” in Results and Fig 2).

$$S_{0i}(t)=(1-k_{i}\times t)$$ (Eq 2)

The fitted k_i_ values (i.e., the survival reduction rates pre-disaster and post-disaster (within 90 days after the disaster)) are shown in Fig 2. All statistical analyses were conducted with IBM SPSS Statics 19 (IBM, New York, USA) or R [13] and its packaged software EZR [14].

#### LLE calculation

LLE calculation due to evacuation is a new concept we developed as part of this study, whereas LLEs due to radiation were estimated on the basis of traditional methods by using cancer risk models and general survival probability (see “Estimation of cancer risks and LLE due to radiation” in Method and “Cancer risk models” in S1 Methods). LLE due to evacuation was estimated by using the survival probability of each subgroup pre- and post-disaster. Namely, we calculated LLEs for 16 subgroups obtained by combining confounding factors (4 for age × 2 for sex × 2 for care level) for both Nursing home group A and Nursing home group B (S2 Fig). After obtaining LLEs for each subgroup, we calculated the sum of LLEs by using the number of residents in each subgroup in Nursing home group A after the disaster. We obtained two sums of LLEs, i.e. LLEs due to evacuation plus non-evacuation-related effects (based on survival data for residents in Nursing home group A) and LLEs due to non-evacuation-related effects (based on those for Nursing home group B). By using the commercially available software Crystal Ball (Oracle, California, USA), we estimated 95% confidential intervals for the LLEs on the basis of k and β coefficients of the Cox proportional hazard regression model through Monte Carlo simulation. Variations in the k and β coefficients of the Cox proportional hazard regression model were assumed to follow a normal distribution. The simulation was performed 10,000 times.

### Estimation of cancer risks and LLE due to radiation

#### Estimation of additional doses

We considered three pathways of exposure to artificial radionuclides released from the Fukushima Daiichi nuclear power plant under Scenarios 1 and 2, namely external exposure, inhalation, and ingestion, in accordance with previous studies [15–17]. In order to focus on the differences in doses in the target periods between Scenarios 1 (rapid evacuation) and 2 (90-day delayed evacuation), we also calculated the additional effective doses and organ doses for nursing home residents and staff between 22 March and 19 June. After the evacuation under Scenario 1, doses were estimated under the assumption that nursing home residents and staff were moved to Kanagawa Prefecture, as described above. The details were described in S1 Methods.

#### Estimation of cancer risks and LLEs

Lifetime attributable risks (LARs) of incidence and mortality and LLEs for all solid cancers and leukemia were estimated for the two sexes and for the age groups. The LARs of cancer incidence were used not to calculate LLEs but as estimated indicators of detriment. For the nursing home staff, ages 20 y, 30 y, 40 y, 50 y, and 60 y in the first year were used to represent ages 19–29 y, 30–39 y, 40–49 y, 50–59 y, and 60–69 y, respectively, whereas for nursing home residents 50 y, 60 y, 70 y, and 80 y were used to represent 50–59 y, 60–69 y, 70–79 y, and ≥80 y, respectively. Because the doses and LARs did not have a linear relationship in linear-quadratic dose-response models, in order to evaluate the differences in LARs between two scenarios the LARs for each scenario should be estimated on the basis of lifetime exposure doses and the differences in LARs should then be calculated. However, because the doses in Scenarios 1 and 2 were small (~1 mSv) and could be therefore regarded to have a linear relationship with the LARs, we used the organ doses from 22 March to 19 June 2011 to focus on the differences in LARs and LLEs among scenarios.

Cancer incidences were estimated up to the age of 89 y in accordance with the method described in a WHO report [2,4] based on the Life Span Study cohort of Hiroshima and Nagasaki atomic bomb survivors, with modification by updating of the mortality and cancer incidence data in Japan [18–20]. Briefly, a linear-quadratic dose-response model was used for leukemia [21] and an linear non-threshold (LNT) model was used for all solid cancers [22]. Note that a mortality model was used for leukemia, but in this regard there was little difference between leukemia incidence and mortality. To correct the risk at low dose levels, in accordance with the ICRP [1], we set a factor of 2 for LNT models (dose and dose-rate effectiveness factor; DDREF) from the perspective of radiological protection. The sum of risks of all solid cancers and leukemia is intended to provide the overall risk of cancer due to radiation exposure; however, the risk of all solid cancers could underestimate the cancer risk in specific tissues such as thyroid cancer in circumstances where the tissue doses are highly heterogeneous [2]. LAR was calculated from cancer-free survival rates (*S*(*a*, *g*)) and a model combining an excess absolute risk (EAR) model and an excess relative risk (ERR) model. Details of the incidence risk models were described in S1 Methods.

Cancer mortalities were estimated from linear-quadratic dose-response models for all solid cancers [23] and leukemia [24,25]. A previous study [23] showed that the linear-quadratic dose-response model provided the better fit in the range of < 2 Gy, whereas the LNT model gave the better fit with in no range limitation. For both all solid cancers and leukemia, ERR models were applied to the recent Life Span Study cohort [23]. Because estimated cancer mortalities do not reflect possible future advances in medical cancer care, the values were considered to have been overestimated. The mortality rates for all solid cancers and leukemia were derived from the age- and sex-stratified all-cause mortality in Japan [18]. Details of the mortality risk models were also described in S1 Methods.

On the basis of the age-specific mortality rates of leukemia and all solid cancers estimated above, LLEs were calculated by using the survival probability for Japanese males and females (S3 Fig) [26]. Because the survival reduction rates for nursing home residents (Fig 2) were larger than those for males and females of the same age in the general Japanese population (S3 Fig), the LLEs for nursing home residents were overestimated. As an example, to evaluate the difference in LLEs between two survival probabilities, instead of the survival probability of the general Japanese population we used the survival probability for a typical subgroup, namely females aged 80–89 y whose care level was “low/moderate,” in Nursing home group A before the disaster (i.e., 50 of 191 nursing home residents were in this subgroup). The LLEs estimated by using the survival probability of the nursing home subgroup were approximately one order of magnitude lower than those obtained by using the survival probability of the general Japanese population. However, this did not influence our conclusions, because the LLEs for nursing home residents due to radiation were much smaller than those for nursing home staff or those due to evacuation. (Total LLEs for nursing home residents due to radiation were approximately one order of magnitude lower than those for nursing home staff; see details in “Risk trade-off between evacuation and radiation” in Results.)

## Results

### Evacuation-related mortality risks

Survival probabilities, adjusted by age, sex, and care level before and after the disaster in Nursing home groups A and B and determined by Cox proportional hazard regression model are shown in Fig 2. S1 Table shows the *β* coefficients of the Cox proportional hazard regression model; survival probabilities of males were significantly lower than those of females and declined with increasing age and care level (*P* < 0.001). The adjusted survival probabilities showed that the declines in survival were almost constant over the whole period before the disaster and for 90 days after the disaster in Nursing home groups A and B (*r* ^2^ = 0.920–0.997, *P* < 0.001). For Nursing home group A, k_i_ (i.e., the survival reduction rates pre-disaster and post-disaster (within 90 days after the disaster) were 0.000293 d^–1^ (95% confidential interval: 0.000290–0.000295 d^-1^) and 0.00238 d^-1^ (0.00227–0.00248 d^-1^), respectively. For Nursing home group B they were 0.000357 d^-1^ (0.000349–0.000364 d^-1^) and 0.000881 d^-1^ (0.000725–0.00104 d^-1^), respectively.

The post-disaster survival rates in Nursing home groups A and B dropped sharply within approximately 90 days after the disaster in comparison with the pre-disaster survival rates. This gap in survival between post- and pre-disaster in Nursing home group A was much larger than that in Nursing home group B, suggesting that there was an increase in mortality risk due to evacuation, rather than simply due to non-evacuation-related effects (e.g., disaster shock).

The estimated LLEs in Nursing home groups A and B for each factor subgroup (4 for age × 2 for sex × 2 for care level) are summarized in Table 2. Reflecting the *β* coefficients of the Cox proportional hazard regression model, the LLEs of males were higher than those of females and increased with increasing age and care level. Large differences were found between Nursing home groups A and B, irrespective of subgroup (e.g., age 40–69, care level low/moderate, male: 35 d (95% confidential interval: 14–78 d) for Nursing home group A and 2.5 d (95% confidential interval: 0.52–7.6 d) for group B), representing the risk associated with evacuation.

**Table 2:** LLEs of nursing home residents due to evacuation or non-evacuation-related effects (e.g., disaster shock), or both. Values in parentheses are 95% confidential intervals.

Adjustment factor			LLE (d)	
Age(at entry)	Care level	Sex	Nursing home group A	Nursing home group B
40–69	Low/moderate	Male	35 (14–78)	2.5 (0.52–7.6)
40–69	Low/moderate	Female	22 (10–45)	1.6 (0.40–4.3)
40–69	High	Male	47 (19–110)	3.4 (0.65–11)
40–69	High	Female	30 (13–63)	2.1 (0.50–6.0)
70–79	Low/moderate	Male	54 (29–95)	4.0 (1.3–9.4)
70–79	Low/moderate	Female	34 (22–53)	2.5 (1.0–5.1)
70–79	High	Male	70 (36–130)	5.4 (1.6–14)
70–79	High	Female	46 (27–76)	3.4 (1.3–7.4)
80–89	Low/moderate	Male	80 (50–130)	6.3 (2.5–13)
80–89	Low/moderate	Female	53 (41–67)	3.9 (2.1–6.7)
80–89	High	Male	100 (61–170)	8.4 (2.9–19)
80–89	High	Female	69 (48–98)	5.3 (2.4–9.9)
90+	Low/moderate	Male	120 (69–190)	9.7 (3.6–21)
90+	Low/moderate	Female	79 (56–110)	6.2 (2.9–11)
90+	High	Male	140 (80–240)	13 (4.2–29)
90+	High	Female	100 (65–150)	8.2 (3.5–17)

### Radiation-related health risks

The additional effective dose for both nursing home residents and staff under Scenario 1 was 0.010 mSv, whereas that under Scenario 2 was 0.40 mSv for nursing home residents and 0.58 mSv for staff (Table 3). The contributions from external exposure were much larger than those from ingestion under Scenario 2; this was consistent with the findings in a previous report [16]. The additional effective doses from the accident before 22 March were 0.47 mSv (external, 0.31 mSv; inhalation, 0.13 mSv; ingestion, 0.03 mSv) for nursing home residents and 0.57 mSv (external, 0.41 mSv; inhalation, 0.13 mSv; ingestion, 0.03 mSv) for staff. Under Scenario 2, the doses between 22 March and 19 June were comparable to those between the accident and 22 March, demonstrating that staying in the contaminated areas increased the radiation risk.

**Table 3:** Radiation doses to nursing home residents and staff in the 90 days after 22 March 2011 (in mSv). Scenario 1, rapid evacuation; Scenario 2, 90-day delayed evacuation.

	Effective dose	Colon	Bone marrow
Rapid evacuation			
External dose (residents)	0.000	0.000	0.000
External dose (staff)	0.000	0.000	0.000
Ingestion dose (residents and staff)	0.010	0.002	0.002
Total (residents)	0.010	0.002	0.002
Total (staff)	0.010	0.002	0.002
90-day delayed evacuation			
External dose (residents)	0.38	0.34	0.33
External dose (staff)	0.56	0.51	0.50
Ingestion dose (residents and staff)	0.02	0.01	0.01
Total (residents)	0.40	0.35	0.34
Total (staff)	0.58	0.52	0.51

LARs of cancer incidence up to 89 y and mortality and LLEs were then estimated for individual age and sex groups under the four scenarios (Table 4 and S2 Table). Under Scenario 2, the maximum LAR of the sum of all solid cancer and leukemia incidences and the maximum estimated mortality from these causes due to radiation exposure occurred in 20-y-old female staff: the values were 7.1 × 10^−5^ and 4.1 × 10^−5^, respectively. The LAR of all solid cancer mortalities in 20-y-old staff was approximately one order of magnitude higher than that of leukemia mortality, and the differences between two became smaller with increasing age at the time of exposure.

**Table 4:** LARs of cancer mortality and LLEs owing to 90-day stays after 21 March 2011 or to 20- or 100-mSv exposure. Ages are representative of age subgroups in the first year after the accident. Scenario 1, rapid evacuation; Scenario 2, 90-day delayed evacuation.

	Rapid evacuation			90-day delayed evacuation			20-mSv exposure			100-mSv exposure		
	LAR of all solid cancer mortality (10^−5^)	LAR of leukemia mortality (10^−5^)	LLE (d)	LAR of all solid cancer mortality (10^−5^)	LAR of leukemia mortality (10^−5^)	LLE (d)	LAR of all solid cancer mortality (10^−5^)	LAR of leukemia mortality (10^−5^)	LLE (d)	LAR of all solid cancer mortality (10^−5^)	LAR of leukemia mortality (10^−5^)	LLE (d)
Nursing home residents												
50 y (M)	0.0046	0.0009	0.00023	0.76	0.18	0.039	44	11	2.3	240	58	12
50 y (F)	0.0053	0.0006	0.00028	0.88	0.11	0.047	51	6.7	2.7	270	36	15
60 y (M)	0.0030	0.0008	0.00014	0.49	0.16	0.023	29	9.6	1.4	150	51	7.3
60 y (F)	0.0034	0.0005	0.00016	0.57	0.10	0.026	33	6.0	1.5	180	32	8.2
70 y (M)	0.0017	0.0007	0.00006	0.28	0.13	0.011	16	7.5	0.66	87	40	3.5
70 y (F)	0.0020	0.0004	0.00008	0.34	0.08	0.013	20	4.8	0.76	100	26	4.1
80 y (M)	0.0007	0.0004	0.00002	0.11	0.07	0.003	6.6	4.4	0.20	35	23	1.1
80 y (F)	0.0009	0.0003	0.00003	0.16	0.05	0.004	9.2	2.9	0.26	49	16	1.4
Nursing home staff												
20 y (M)	0.013	0.0011	0.00067	3.2	0.33	0.17	130	13	6.6	660	70	35
20 y (F)	0.016	0.0007	0.00094	3.9	0.21	0.23	150	8.4	9.2	810	45	49
30 y (M)	0.0092	0.0011	0.00047	2.3	0.30	0.12	89	12	4.7	470	65	25
30 y (F)	0.011	0.0007	0.00066	2.8	0.19	0.16	110	7.7	6.4	580	41	34
40 y (M)	0.0066	0.0010	0.00034	1.6	0.29	0.086	64	12	3.4	340	62	18
40 y (F)	0.0079	0.0006	0.00044	1.9	0.18	0.11	76	7.3	4.4	400	39	23
50 y (M)	0.0046	0.0009	0.00023	1.1	0.27	0.059	44	11	2.3	240	58	12
50 y (F)	0.0053	0.0006	0.00028	1.3	0.17	0.070	51	6.7	2.7	270	36	15
60 y (M)	0.0030	0.0008	0.00014	0.74	0.24	0.035	29	9.6	1.4	150	51	7.3
60 y (F)	0.0034	0.0005	0.00016	0.84	0.15	0.039	33	6.0	1.5	180	32	8.2

The maximum age-specific rates of mortality from all solid cancers in nursing home staff aged 20 y at the time of exposure showed a unimodal distribution with peaks at 80 y for males and 85 y for females, whereas those of leukemia showed bimodal distributions with peaks at 22 y and 75 y for males and at 25 y and 80 y for females (S4 Fig). The maximum age-specific mortality rates from all solid cancers and leukemia combined were 0.14 × 10^−5^ y^–1^ at age 80 y for males and 0.15 × 10^−5^ y^–1^ at age 85 y for females; these were three orders of magnitude lower than the Royal Society’s acceptable risk level for individuals (10^−3^ y^–1^) [9]. This level was used as a reference by the ICRP in establishing constraints for occupational exposure and eventually in establishing the lowest reference levels in the effective dose bands in emergency exposure situations.

The LLEs for 20-y-old females under Scenarios 1 and 2 were 0.00094 d and 0.23 d, respectively. The LLEs due to only all solid cancers and only leukemia for 20-y-old females under Scenario 2 were 0.22 d and 0.018 d, respectively. The LLEs per one mortality due to all solid cancers, leukemia, and the two combined under Scenario 2 were 15 y, 23 y, and 16 y, respectively, for 20-y-old females. The larger LLEs per one mortality due to leukemia were attributable to the younger mortality peak than with all solid cancers, as described before. However, because the mortality rate from leukemia was more than one order magnitude lower than that from all solid cancers, the contribution of LLE due to leukemia mortality to the total (0.018/0.23) was much smaller than that due to all solid cancers (0.22/0.23).

The LLEs for 20–60-y-olds under the 20-mSv and 100-mSv exposure scenarios were 1.4 to 9.2 d and 7.3 to 49 d, respectively. LLEs due to lifetime exposure to 13 environmental pollutants in Japan are in the range of 0.009 to 14 d, with a maximum in the case of exposure to diesel exhaust particles [27]. The LLEs due to 100-mSv exposure were comparable to, or much higher than, those due to lifetime exposure to environmental pollutants, highlighting the substantial impact of radiation risk due to the nuclear power plant accident.

### Risk trade-off between evacuation and radiation

On the basis of the population distributions of nursing home residents and staff in Nursing home group A and the LLEs associated with evacuation-related risks and radiation risks, we calculated the total LLEs of nursing home residents and staff (Table 5). The total LLE due to evacuation-related risk under Scenario 1 was estimated from the number of residents and the individual LLEs obtained for Nursing home group A to be 11,000 persons-d (95% confidential interval: 10,000–13,000 persons-d). This evacuation-related risk may have included non-evacuation-related effects (e.g. disaster shock); in this case the LLE was calculated to be 880 persons-d (95% confidential interval: 730–1200 persons-d) by using the values for Nursing home group B. In contrast, the total LLEs due to radiation risk under Scenarios 1 and 2 were 0.11 persons-d and 27 persons-d, respectively, showing that the effect of evacuation on reduction of LLE due to radiation was 27 persons-d. Note that evacuation-related risks were estimated in such a way that they were not overestimated, whereas radiation-related risks were not underestimated (see details in Methods). Nevertheless, the total LLE due to evacuation-related risk under Scenario 1 was two to three orders of magnitude higher than that due to the radiation avoided by evacuation. It was also one order of magnitude higher than the LLE due to 20-mSv exposure and double that of the LLE due to 100-mSv exposure, clearly highlighting the prominent risks of evacuation.

**Table 5:** Comparison of LLEs of residents and staff in nursing homes among rapid and 90-day delayed evacuation scenarios and 20-mSv- and 100-mSv exposure scenarios (persons-d). Scenario 1, rapid evacuation; Scenario 2, 90-day delayed evacuation. Total LLEs of evacuation-related risk in Scenario 1 were much higher than those of avoidable risks due to radiation exposure in the other scenarios. Values in parentheses are 95% confidential intervals.

	Rapid evacuation	90-day delayed evacuation	20-mSv exposure	100-mSv exposure
Evacuation-related				
Nursing home residents	11000 (10000–13000)[880 (730–1200)]^a^	Unknown	-	-
Nursing home staff	Not observed	Unknown	-	-
Radiation-related				
Nursing home residents	0.01	1.7	100	530
Nursing home staff	0.1	26	1000	5300
Total	11000+ (10000+–13000+)	27+	1100	5800

^a^ LLEs due to non-evacuation-related effects (e.g. disaster-shock), as estimated from the data from Nursing home group B.

Total LLEs due to radiation for nursing home staff were approximately one order of magnitude higher than those for nursing home residents. Because the number of nursing home staff (184) was similar to that of nursing home residents (191), this difference was attributed mainly to the differences in individual LLEs of each subgroup (Table 4), which in turn came from the differences in both ages and radiation doses between nursing home residents and staff.

## Discussion

Various indicators of risk are used to meet the objectives of studies. For example, in 1990, the ICRP used various detriments, including LAR, annual distribution of LARs, the increase in age-specific mortality rate, and LLE to establish constraints for occupational exposure [8]. In its 2007 recommendations, nominal risks adjusted by mortality rates and quality of life were used as indicators of risk [1]. Other indicators such as quality-adjusted life years (QALY) or disability-adjusted life years (DALY) are often used to rank various risks or to demonstrate trade-offs or the benefits vs. costs of risks [28,29]. Among the various indicators of risks, we used LLE here to compare the risks between evacuation and exposure to radiation. Excess mortality is not an appropriate indicator because of differences in the types of mortality between evacuation and radiation exposure: excess mortality from radiation is late or delayed and occurs over a lifetime, whereas excess mortality from evacuation is acute and occurs over short periods. Excess incidence is also inappropriate, because of the absence of measurable values related to the evacuation. Although quality of life (QOL) can be considered to drop after evacuation, QALY or DALY is also not applicable owing to a lack of quantitative data on QOL reduction. In contrast, the use of LLEs, which have historically been adopted for risk-tradeoff analysis, has advantages. LLEs can be quantitatively estimated on the basis of measurable data. Differences in the time of occurrence of mortality between evacuation and exposure to radiation can be expressed in consideration of the age reached at the time of death, allowing us to compare the risks between evacuation and radiation exposure. Although it could be argued that it is appropriate to weight the life expectancy days of nursing home residents and staff differently, in our study we considered that life expectancy days had the same weight for all individuals, irrespective of age or state of health.

As shown in Table 5, evacuation-related risk was serious for nursing home residents, whereas the radiation-related risk was biased toward nursing home staff. As a result, adopting Scenario 2 instead of Scenario 1 would reduce the total LLE, but it would increase the LLE for nursing home staff. So it seems that the risk trade-off here includes the question of equity or fairness of risk distribution among interest groups. Note, however, that the evacuation-related risk for nursing home staff was not evaluated here. Evacuation can have adverse health effects on nursing home staff, as has been found among general evacuees [5,6], but we did not capture these risks in our study. It is not clear whether or not the risk to staff would really increase in Scenario 2 in comparison with Scenario 1. It remains a matter of debate whether evacuation increased the overall risk to nursing home staff.

The decision to evacuate depends on the anticipated radiation dose and the situation. The total LLEs estimated under the two evacuation scenarios and the 20-mSv exposure scenario demonstrated that the evacuation-related risk to nursing home residents was much higher than the avoidable risk due to radiation at general dose levels. The evacuation-related risk was still higher than the risk due to exposure to radiation at 100 mSv, which, here, was estimated on the basis of a linear-quadratic cancer mortality model. Here, our intention is not to insist that the decisions made by nursing homes in the 2011 accident were inappropriate. The radiation status, nuclear power plant control, and medical resources were not clear at the time, and concern about the deteriorating situation was increasing. Emergency planning for evacuation had not been decided on, and blame could therefore not be apportioned for the decision to evacuate. Notably, the evacuation-related risks under Scenario 2 were not quantified. In general, decisions in times of emergency are always difficult owing to lots of unknown factors. Note, however, the ICRP [1] recommendation that a rise in dose towards 100 mSv will almost always justify protective measures and that evacuation-related risks are higher than the risks from exposure to radiation at 100 mSv. There needs to be serious deliberation as to whether to reduce evacuation-related risks or pursue protective actions other than evacuation. Our intention here was to demonstrate that, as part of emergency preparedness, ways of reducing evacuation-related risks should be fully considered before accidents. Our risk trade-off analysis highlighted the importance of understanding and preparing for evacuation-related risks.

Evacuation-related risks were likely attributable to the combined effects of physical stress due to movement, limitations on medical resources, and changes in medical staff [10–12]. The increase in mortality rate was not significantly correlated with movement distance or the number of repeat evacuations [10]. The contribution of physical stress due to movement might be related to the manner of evacuation, including the level of care received during movement. After the accident, these radiation-risk areas were isolated and had insufficient medical resources. Changes in medical staff may have been among the factors related to the increase in mortality risks after the evacuation, because the medical staff who initially cared for the nursing home residents might have had difficulty in continuing that care at evacuation sites. Preparation of medical protocols or enhancement of communication among staff might have mitigated the increased mortality risks. However, even if nursing home residents could have been evacuated smoothly and without stress or medical problems related to resources and staff, other, non-evacuation-related, risks, including the effects of disaster shock, were likely inevitable. On the other hand, staying without evacuation for a longer time might have placed the residents at additional risk because of increased physical or mental stress and limitations on drug supplies.

Again, our emphasis here is that the latitude for reducing evacuation risk is surprisingly large. The benefit of such a reduction is larger than, or comparable to, the 100-mSv exposure risk, although the evacuation plans provided by the government still lacked careful consideration of medical care during and after the evacuation. The most important points are that we need to take evacuation-related risk into account together with radiation exposure risk, and that we need to improve our social system in order to mitigate evacuation-related risks. Identification of the causes of evacuation mortality and development of mitigative evacuation planning are now justified. In addition, compulsory evacuation needs to be well balanced with the trade-off against radiation risk and in consideration of the concept of acceptable risk. Comprehensive strategies that fully consider both radiation risks and evacuation-related risks will minimize the overall risk to society.

## Supporting Information

S1 Fig (a) Locations of Fukushima Prefecture, Tokyo, and Kanagawa Prefecture. (b) Locations of the cities of Minamisoma and Soma, and classification of areas for evacuation (22 April 2011). (PDF)

S2 Fig Estimated pre- and post-disaster survival of each subgroup in Nursing home groups A and B. Age (at entry), care level, and sex are as follows: (a) 40–69, Low/moderate, Male; (b) 40–69, Low/moderate, Female; (c) 40–69, High, Male; (d) 40–69, High, Female; (e) 70–79, Low/moderate, Male; (f) 70–79, Low/moderate, Female; (g) 70–79, High, Male; (h) 70–79, High, Female; (i) 80–89, Low/moderate, Male; (j) 80–89, Low/moderate, Female; (k) 80–89, High, Male; (l) 80–89, High, Female; (m) 90+, Low/moderate, Male; (n) 90+, Low/moderate, Female; (o) 90+, High, Male; (p) 90+, High, Female.
(PDF)

S3 Fig Survival probabilities of Japanese males and females. (PDF)

S4 Fig Attributable age-specific mortality rates due to radiation exposure under Scenario 2 (age 20 y at time of exposure). (a) All solid cancers, (b) leukemia, (c) Combination of all solid cancers and leukemia. Data were plotted at 5-y intervals.
(PDF)

S5 Fig Kerma rates in free air, as surveyed by a monitoring post in Minamisoma. (a) From 11 March to 1 July 2011, (b) from 11 March to 25 March 2011.
(PDF)

S1 Methods Estimation of additional effective doses and cancer risk models. (PDF)

S1 Table Estimated parameters of the Cox proportional hazard regression model. (PDF)

S2 Table LARs of cancer incidence up to 89 y resulting from stays for different lengths of time in the 90 days after 21 March 2011 or from exposure to 20 or 100 mSv (10^−5^). Ages are representative of age subgroups in the first year after the accident. Scenario 1, rapid evacuation; Scenario 2, 90-day delayed evacuation.
(PDF)
