# Supplementary material for: Was the Risk from Nursing-Home Evacuation after the Fukushima Accident Higher than the Radiation Risk?
Source: PLoS One. 2015 Sep 11;10(9):e0137906. doi: 10.1371/journal.pone.0137906 (PMC4567272; doi:10.1371/journal.pone.0137906)
Supplement: S1 Fig — (PDF) [file pone.0137906.s001.pdf]

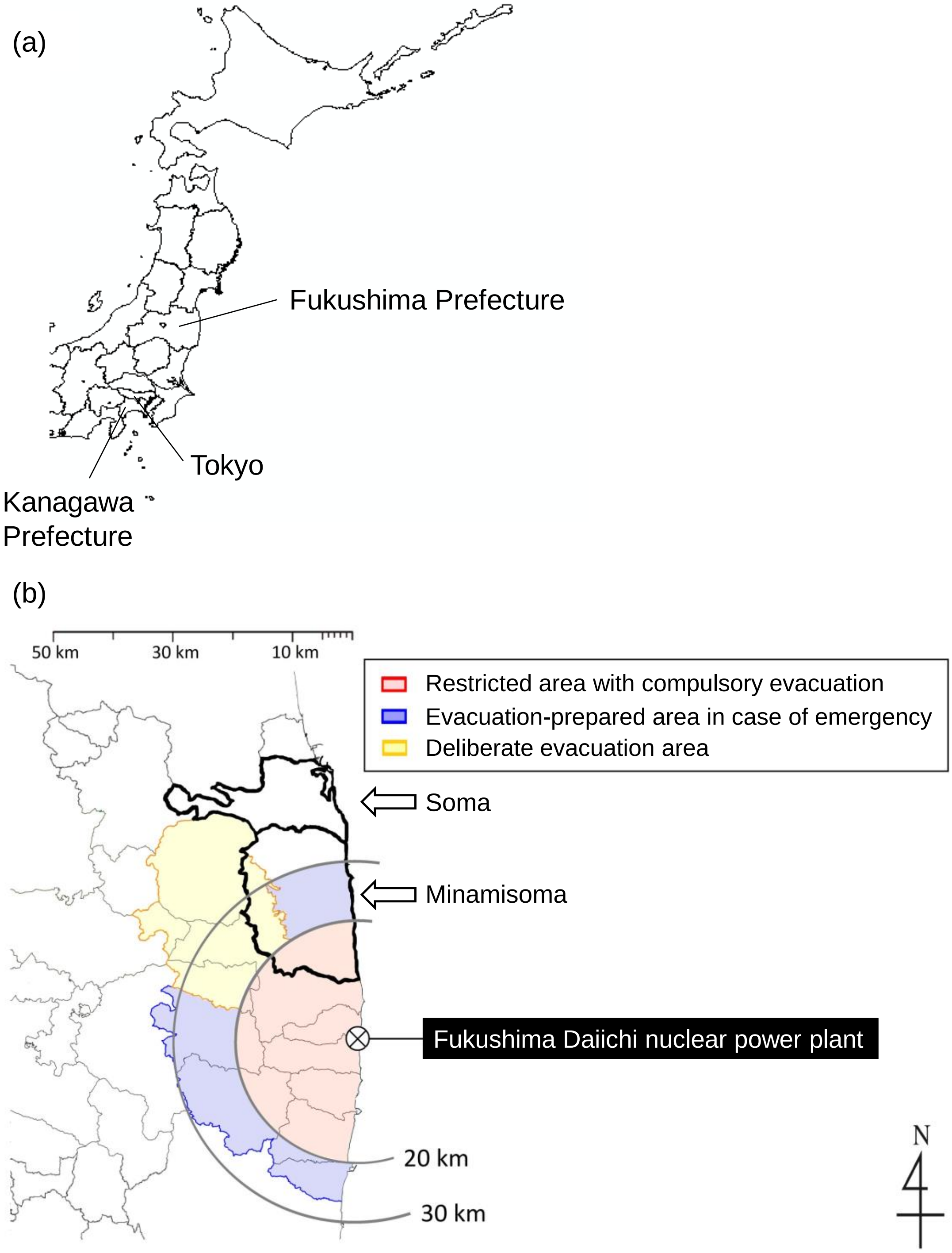

S1 Fig. (a) Locations of Fukushima Prefecture, Tokyo, and Kanagawa Prefecture. (b) Locations of the cities of Minamisoma and Soma, and classification of areas for evacuation (22 April 2011).
