# Supplementary material for: Was the Risk from Nursing-Home Evacuation after the Fukushima Accident Higher than the Radiation Risk?
Source: PLoS One. 2015 Sep 11;10(9):e0137906. doi: 10.1371/journal.pone.0137906 (PMC4567272; doi:10.1371/journal.pone.0137906)
Supplement: S2 Fig — Age (at entry), care level, and sex are as follows: (a) 40–69, Low/moderate, Male; (b) 40–69, Low/moderate, Female; (c) 40–69, High, Male; (d) 40–69, High, Female; (e) 70–79, Low/moderate, Male; (f) 70–79, Low/moderate, Female; (g) 70–79, High, Male; (h) 70–79, High, Female; (i) 80–89, Low/moderate, Male; (j) 80–89, Low/moderate, Female; (k) 80–89, High, Male; (l) 80–89, High, Female; (m) 90+, Low/moderate, Male; (n) 90+, Low/moderate, Female; (o) 90+, High, Male; (p) 90+, High, Female. (PDF) [file pone.0137906.s002.pdf]

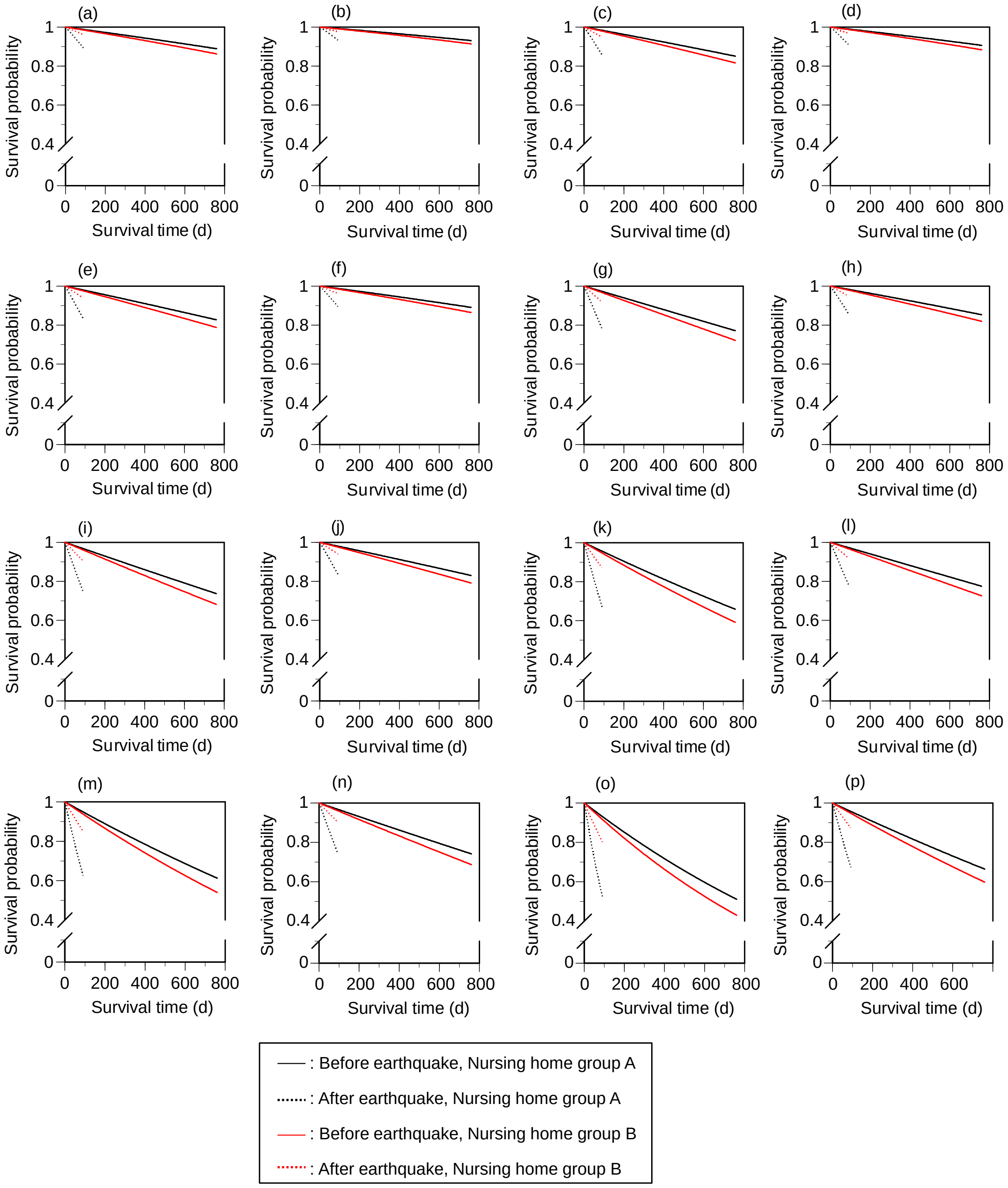

S2 Fig. Estimated pre- and post-disaster survival of each subgroup in Nursing home groups A and B. Age (at entry), care level, and sex are as follows: (a) 40–69, Low/moderate, Male; (b) 40–69, Low/moderate, Female; (c) 40–69, High, Male; (d) 40–69, High, Female; (e) 70–79, Low/moderate, Male; (f) 70–79, Low/moderate, Female; (g) 70–79, High, Male; (h) 70–79, High, Female; (i) 80–89, Low/moderate, Male; (j) 80–89, Low/moderate, Female; (k) 80–89, High, Male; (l) 80–89, High, Female; (m) 90+, Low/moderate, Male; (n) 90+, Low/moderate, Female; (o) 90+, High, Male; (p) 90+, High, Female.
