# Supplementary figures and images for: Was the Risk from Nursing-Home Evacuation after the Fukushima Accident Higher than the Radiation Risk?
Source: PLoS One. 2015 Sep 11;10(9):e0137906. doi: 10.1371/journal.pone.0137906 (PMC4567272; doi:10.1371/journal.pone.0137906)

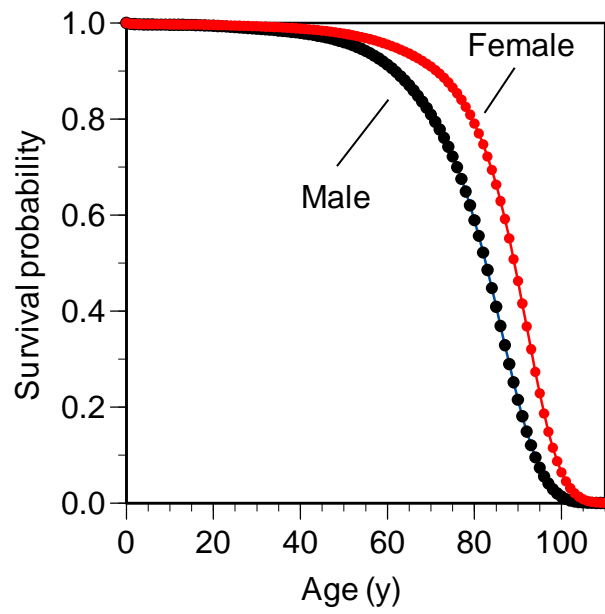

S3 Fig. Survival probabilities of Japanese males and females.

Supplement: S3 Fig — (PDF) [file pone.0137906.s003.pdf]
