# Supplementary material for: Was the Risk from Nursing-Home Evacuation after the Fukushima Accident Higher than the Radiation Risk?
Source: PLoS One. 2015 Sep 11;10(9):e0137906. doi: 10.1371/journal.pone.0137906 (PMC4567272; doi:10.1371/journal.pone.0137906)
Supplement: S4 Fig — (a) All solid cancers, (b) leukemia, (c) Combination of all solid cancers and leukemia. Data were plotted at 5-y intervals. (PDF) [file pone.0137906.s004.pdf]

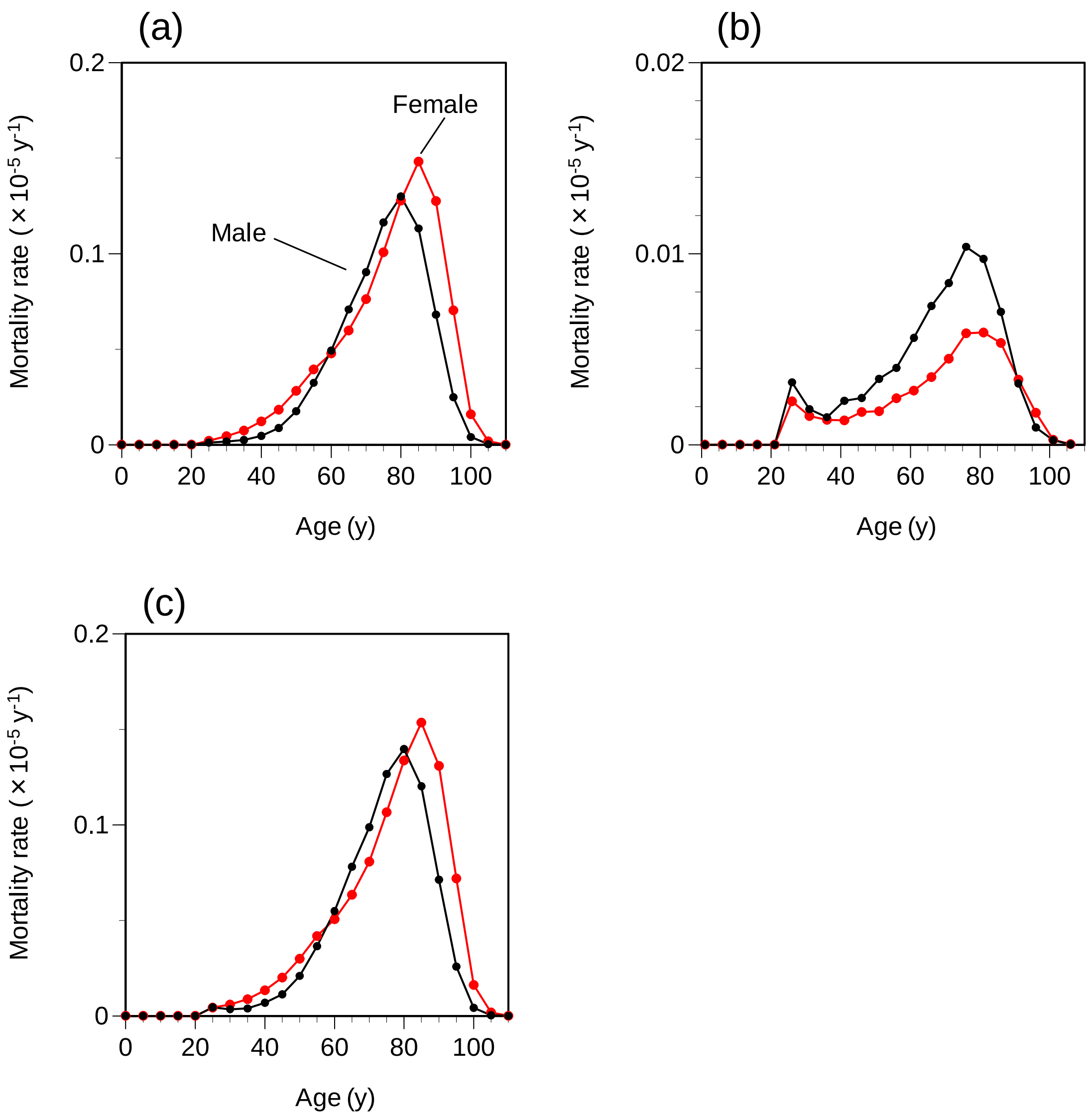


S4 Fig. Attributable age-specific mortality rates due to radiation exposure under Scenario 2 (age 20 y at time of exposure). (a) All solid cancers, (b) leukemia, (c) Combination of all solid cancers and leukemia. Data were plotted at 5-y intervals.
