# Supplementary material for: Was the Risk from Nursing-Home Evacuation after the Fukushima Accident Higher than the Radiation Risk?
Source: PLoS One. 2015 Sep 11;10(9):e0137906. doi: 10.1371/journal.pone.0137906 (PMC4567272; doi:10.1371/journal.pone.0137906)
Supplement: S5 Fig — (a) From 11 March to 1 July 2011, (b) from 11 March to 25 March 2011. (PDF) [file pone.0137906.s005.pdf]

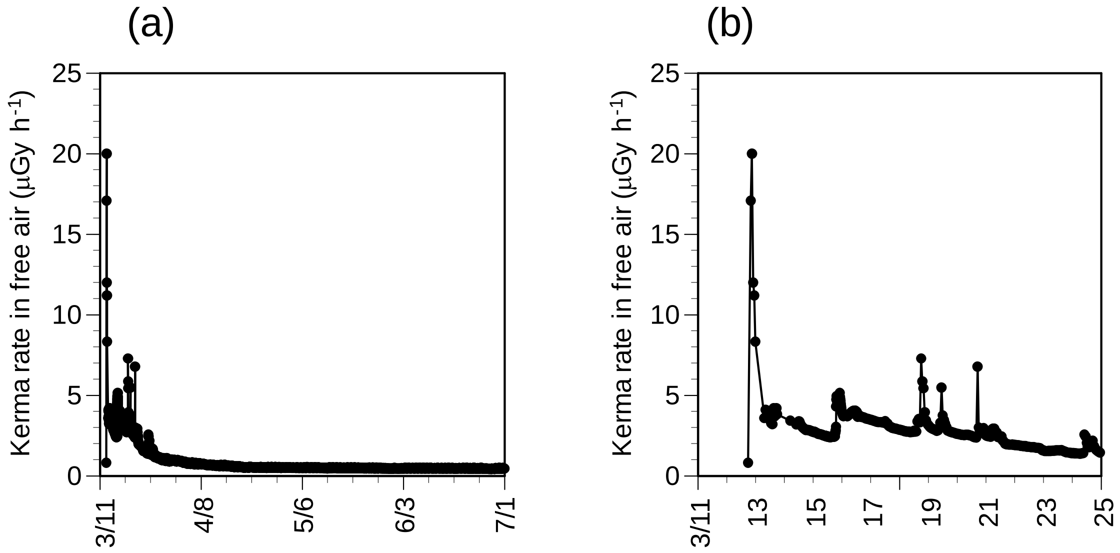

S5 Fig. Kerma rates in free air, as surveyed by a monitoring post in Minamisoma. (a) From 11 March to 1 July 2011, (b) from 11 March to 25 March 2011.
