# Supplementary material for: Was the Risk from Nursing-Home Evacuation after the Fukushima Accident Higher than the Radiation Risk?
Source: PLoS One. 2015 Sep 11;10(9):e0137906. doi: 10.1371/journal.pone.0137906 (PMC4567272; doi:10.1371/journal.pone.0137906)
Supplement: S1 Methods — (PDF) [file pone.0137906.s006.pdf]

S1 Methods

Estimation of additional effective doses

The additional effective dose from external exposure was estimated as follows [1]:

$$D_{ee} = \int_{st}^{et} (ADR \times k - BGR) dt \times (t_{out} + t_{in} \times RF)/24 \qquad \text{[eq.S1]}$$

where D_{ee} is the additional effective dose from external exposure [Sv], st is the start of the date in the scenario, et is the end of the date in the scenario, ADR is the kerma rate in free air [Gy/h], k is the conversion coefficient from the kerma rate in free air to the effective dose [Sv/Gy], BGR is the background rate [0.03 μSv/h] [1], t_{out} is the time spent outside buildings per day[h], t_{in} is the time spent inside buildings per day, and RF is the reduction factor.

ADR followed the monitoring data, as determined by a fixed radiation monitoring post in the Minamisoma Joint Government building in Minamisoma [2] (Figure S5). This included all radiation exposure to radioiodine and radiocesium from cloud shine and ground shine; the radiation peaked at 21:00 on 12 March and then decreased with time. We used a k value of 0.75 to estimate the doses in Scenarios 1 and 2 [3,4]. RF was set at 0.9 for cloud shine before 14 March, whereas RF was set at 0.4 for ground shine for the period from 14 March onward [1]. The RF value of 0.4 for ground shine was comparable to the average value of 0.34 observed in households in Fukushima Prefecture [5]. For nursing home staff, t_{out} and t_{in} were set at 8 h and 16 h,

respectively [4], whereas for nursing home residents, t_{out} and t_{in} were set at 0 h and 24 h. The external exposure dose at the evacuation site was regarded as negligible, thus also resulting in overestimation of the radiation risks avoided after evacuation.

Because the radioactive plume passed before the start date of the scenarios (22 March), doses from inhalation were not included in the estimation of LLEs in either of the evacuation scenarios. To compare the dose levels before 22 March with those in the subsequent 90 d, the doses were approximated as follows [4]:

$$D_{ih} = A_{Cs137} \times I_i \times \sum_m (A_m / A_{cs137}) / V_{bm} \times d_{mi} \qquad \text{[eq.S2]}$$

Where D_{ih} is the additional effective dose from inhalation [Sv], m is the radionuclide, A_m is the surface activity density of radionuclide m on the ground [Bq m^{-2}], I_i is the breathing rate [m^3 s^{-1}], V_{bm} is the bulk deposition velocity of radionuclide m [m s^{-1}], and d_{mi} is the effective dose inhalation coefficient for radionuclide m [Sv Bq^{-1}]. The median of measured values (99800 Bq m^{-2} [6]) in the city of Minamisoma was used for A_{Cs137}. Radionuclides ^{131}I, ^{132}I, ^{132}Te, ^{134}Cs, and ^{137}Cs were considered in the assessment.

Doses from ingestion in Minamisoma were assumed to be similar to those in the city of Fukushima, whereas ingestion doses at the evacuation site (in Kanagawa Prefecture, the prefecture neighboring Tokyo) were assumed to similar to those in

Tokyo. The doses followed arithmetic means for males and females ≥19 y old [7]. In this regard, however, for doses due to ingestion of drinking water at the evacuation site, we used monitoring data from the Water Purification Plant in Kanagawa Prefecture [8].

Cancer risk models

Lifetime attributable risk (LAR) was calculated from cancer-free survival rates ($S(a, g)$) and a model combining an excess absolute risk (EAR) model and an excess relative risk (ERR) model.

$$\mathrm{LAR}\ (D, e, g) = \int_{e+L}^{89} [w \cdot \mathrm{EAR}(D, e, a, g) + (1 - w)\ \times\ \mathrm{ERR}(D, e, a, g)\ \times\ m(a, g)] \frac{S(a,g)}{S(e,g)} da$$

[eq.S3]

where D is the organ dose (Sv), e is age at exposure, g is sex, L is the minimum latency period (2 y for leukemia and 5 y for all solid cancers), w is the weight of the EAR and ERR models combined (0.5), a is the age attained, and $m(a, g)$ is the baseline cancer incidence in the unexposed population. For Scenario 1 and 2, the organ (colon and bone marrow) doses were calculated from the additional effective doses and the organ dose to effective dose ratios [9]. For the exposure scenarios, 20-mSv or 100-mSv organ doses were used in the assessment.

Incidence risk models for all solid cancers were as follows:

EAR (D, e, a, g) or ERR $(D, e, a, g) = (1 + t \times s) \times k_d \times D \times \exp[-g_e \times (e\text{-}30) + g_a \times \ln(a/70)]$ [eq.S4]

where, s = –1 for males and 1 for females and, for the EAR model, $t = 0.1622$, $k_d = 51.63 \times 10^{-4}$, $g_e = 0.02805$, and $g_a = 2.406$; for the ERR model, $t = 0.2465$, $k_d = 0.4666$, $g_e = 0.01849$, and $g_a = -1.621$.

The incidence risk models and their parameters for leukemia were as follows:

EAR $(D, e, a, g) = (\alpha \times D + \beta \times D^2) \times \exp[\kappa_1 \times l_{s=female} + \kappa_2 \times \ln(a - e)]$

[eq.S5]

where $\alpha = 7.5165 \times 10^{-4}$, $\beta/\alpha = 1.03455$, $\kappa_1 = -0.52526$, $\kappa_2 = -0.6141$, and $l_{s=female} = 0$ for males, 1 for females.

ERR $(D, e, a, g) = (\alpha \times D + \beta \times D^2) \exp[\kappa_1 \times \ln(a)]$ [eq.S6]

where $\alpha = 864.552$, $\beta/\alpha = 1.18092$, $\kappa_1 = -1.647$.

The mortality risk model for all solid cancers was as follows:

ERR $(D, e, a, g) = (\alpha \times D + \beta \times D^2) \times (1 + t \times s) \times \exp[g_e \times (e\text{-}30) + g_a \times \ln(a/70)]$

[eq.S7]

where α = 0.22, β = 0.18, t = 0.29, g_e = –0.034, and g_a = –0.89.

The mortality risk model for leukemia was as follows:

$$\mathrm{ERR}\,(D, a) = (\alpha \times \mathrm{D} + \beta \times \mathrm{D}^2) \times \exp[\gamma \times \ln[a/50]] \qquad \text{[eq.S8]}$$

where α = 1.612, β = 1.551, and γ = –1.634.

References

1. Akahane K, Yonai S, Fukuda S, Miyahara N, Yasuda H, Iwaoka K, et al. (2013) NIRS external dose estimation system for Fukushima residents after the Fukushima Dai-ichi NPP accident. Sci Rep 3: 1670.

2. Fukushima Prefecture (2011) Results of radiation monitoring in the past: http://www.pref.fukushima.lg.jp/sec/16025d/kako-monitoring.html. Accessed: Sep. 2014 [in Japanese]

3. Golikov V, Wallstroem E, Woehni T, Tanaka K, Endo S, Hoshi M (2007) Evaluation of conversion coefficients from measurable to risk quantities for external exposure over contaminated soil by use of physical human phantoms. Radiat Environ Bioph 46: 375-382.

4. World Health Organization (2012) Preliminary dose estimation from the nuclear accident after the 2011 Great East Japan Earthquake and Tsunami.

5. Takahara S, Iijima M, Shimada K, Kimura M, Homma T (2014) Chapter 18, probabilistic assessment of doses to the public living in areas contaminated by the Fukushima Daiichi nuclear power plant accident. In Radiation monitoring and dose estimation of the Fukushima nuclear accident; Takahashi S, editor: Springer Japan.
6. Ministry of Education Culture Sports Science and Technology (2011) Results of radionuclides (^{134}Cs and ^{137}Cs) in soils: http://www.mext.go.jp/b_menu/shingi/chousa/gijyutu/017/shiryo/__icsFiles/afieldfile/2011/09/02/1310688_1.pdf. Accessed: Sep. 2014 [in Japanese]
7. Murakami M, Oki T (2014) Estimated dietary intake of radionuclides and health risks for the citizens of Fukushima City, Tokyo, and Osaka after the 2011 nuclear accident. PLOS ONE 9: e112791.
8. Kanagawa Prefectural Government (2014) Surveys of radionuclides in tap water in Kanagawa Prefectural Institute of Public Health: http://www.pref.kanagawa.jp/uploaded/attachment/450333.pdf. Accessed: Sep. 2014 [in Japanese]
9. World Health Organization (2013) Health risk assessment from the nuclear accident after the 2011 Great East Japan Earthquake and Tsunami based on a preliminary dose estimation.
