# Supplementary material for: Was the Risk from Nursing-Home Evacuation after the Fukushima Accident Higher than the Radiation Risk?
Source: PLoS One. 2015 Sep 11;10(9):e0137906. doi: 10.1371/journal.pone.0137906 (PMC4567272; doi:10.1371/journal.pone.0137906)
Supplement: S1 Table — (PDF) [file pone.0137906.s007.pdf]

S1 Table. Estimated parameters of the Cox proportional hazard regression model.

n	β_n [a]		P
1 (age at entry)	0.47	(0.35 – 0.60)	< 0.001
2 (care level)	0.31	(0.13 – 0.50)	< 0.001
3 (sex)	-0.49	(-0.70 – -0.28)	< 0.001

[a] Values in parentheses represent 5th–95th percentiles.
