# Supplementary material for: Was the Risk from Nursing-Home Evacuation after the Fukushima Accident Higher than the Radiation Risk?
Source: PLoS One. 2015 Sep 11;10(9):e0137906. doi: 10.1371/journal.pone.0137906 (PMC4567272; doi:10.1371/journal.pone.0137906)
Supplement: S2 Table — Ages are representative of age subgroups in the first year after the accident. Scenario 1, rapid evacuation; Scenario 2, 90-day delayed evacuation. (PDF) [file pone.0137906.s008.pdf]

S2 Table. LARs of cancer incidence up to 89 y resulting from stays for different lengths of time in the 90 days after 21 March 2011 or from exposure to 20 or 100 mSv (10^{-5}). Ages are representative of age subgroups in the first year after the accident. Scenario 1, rapid evacuation; Scenario 2, 90-day delayed evacuation.

	Rapid evacuation		90-day delayed evacuation		20-mSv exposure		100-mSv exposure	
	All solid cancers	Leukemia	All solid cancers	Leukemia	All solid cancers	Leukemia	All solid cancers	Leukemia
Nursing home residents								
50 y (M)	0.0079	0.0008	1.3	0.15	75	8.8	370	48
50 y (F)	0.010	0.0005	1.7	0.10	99	6.0	490	32
60 y (M)	0.0051	0.0006	0.84	0.12	48	7.1	240	39
60 y (F)	0.0065	0.0004	1.1	0.08	62	4.9	310	26
70 y (M)	0.0026	0.0005	0.43	0.09	25	5.2	120	29
70 y (F)	0.0034	0.0003	0.56	0.06	32	3.6	160	19
80 y (M)	0.0007	0.0003	0.12	0.05	6.8	3.0	34	16
80 y (F)	0.0010	0.0002	0.16	0.03	9.4	1.9	47	11
Nursing home staff								
20 y (M)	0.019	0.0012	4.6	0.34	180	14	880	74
20 y (F)	0.028	0.0008	6.9	0.23	270	9.1	1300	49
30 y (M)	0.014	0.0010	3.6	0.29	140	12	680	64
30 y (F)	0.021	0.0007	5.2	0.20	200	7.9	1000	43
40 y (M)	0.011	0.0009	2.7	0.26	100	10	520	56
40 y (F)	0.015	0.0006	3.8	0.17	150	6.9	730	38
50 y (M)	0.0079	0.0008	1.9	0.22	75	8.8	370	48
50 y (F)	0.010	0.0005	2.6	0.15	99	6.0	490	32
60 y (M)	0.0051	0.0006	1.3	0.18	48	7.1	240	39
60 y (F)	0.0065	0.0004	1.6	0.12	62	4.9	310	26
